# Investigating the Mechanism of Rhizoma Coptidis-Eupatorium fortunei Medicine in the Treatment of Type 2 Diabetes Based on Network Pharmacology and Molecular Docking

**DOI:** 10.1155/2022/7978258

**Published:** 2022-11-21

**Authors:** Huan Li, Dan Luo, Ran Wei, Mingliang Sun, Xi Zhang, Huan Deng, Wenfei Bian, Haoyue Wei, Yanqin Huang

**Affiliations:** ^1^First Clinical Medical College, Shandong University of Traditional Chinese Medicine, Jinan 250014, China; ^2^Department of Endocrinology, Affiliated Hospital of Shandong University of Traditional Chinese Medicine, Jinan 250014, China; ^3^Department of Science and Technology, Affiliated Hospital of Shandong University of Traditional Chinese Medicine, Jinan 250014, China; ^4^College of Traditional Chinese Medicine, Shandong University of Traditional Chinese Medicine, Jinan 250014, China

## Abstract

**Objective:**

This study systematically explored the mechanism of Rhizoma Coptidis-Eupatorium fortunei in treating type 2 diabetes mellitus (T2DM) by using network pharmacology and molecular docking methods.

**Methods:**

The TCMSP database was used to screen out the active ingredients and related targets of Rhizoma Coptidis-Eupatorium fortunei (R-E) drug pair. GeneCards, OMIM, DrugBank, and other databases were used to screen the related targets of T2DM, and then, the UniProt database was used to standardize the relevant targets of T2DM. Then, the Venn analysis was performed on the active ingredient-related targets and disease-related targets of R-E drugs to find the intersection targets. Using the STRING database and Cytoscape software, the PPI network and “drug-active ingredient-target-disease” network are constructed by intersecting targets and corresponding active ingredients. Through the cluster profiler package in the R software, GO function enrichment analysis and KEGG pathway enrichment analysis were carried out on the intersection targets and the screened core targets, and the prediction results were verified by molecular docking.

**Results:**

Taking OB ≥ 30% and DL ≥ 0.18 as the standard, a total of 25 effective active ingredients of R-E drug pairs were screened, including berberine, palmatine, coptisine, and so on. After corresponding, 19 effective chemical components and 284 targets of the R-E drug pair were obtained. After searching multiple disease databases, 1289 T2DM-related targets were screened. After the summary, 159 common targets were obtained in this study. Finally, in the bioinformatics analysis, this study concluded that quercetin, luteolin, berberine, palmatine, and coptisine are the main chemical components of the R-E drug pair. ESR1, MAPK1, AKT1, TP53, IL6, and JUN are the important core targets. GO and KEGG enrichment analyses showed that Rhizoma Coptidis-Eupatorium fortunei could improve T2DM by regulating multiple biological processes and pathways. Molecular docking results showed that berberine, palmatine, and coptisine had higher binding to the core target, and MAPK1, AKT1, and IL6 could stably bind to the active ingredients of Rhizoma Coptidis-Eupatorium fortunei.

**Conclusion:**

Rhizoma Coptidis-Eupatorium fortunei may have therapeutic effects on T2DM such as anti-inflammatory and regulating glucose and lipid metabolism through multiple components, multiple targets, and multiple signaling pathways, which provides a scientific basis for further research on the hypoglycemic effect of Rhizoma Coptidis-Eupatorium fortunei drug pair.

## 1. Introduction

Type 2 diabetes mellitus (T2DM) is a chronic metabolic disease characterized by polydipsia, polyphagia, polyuria, and weight loss as the main clinical manifestations [1]. The main pathological factors of T2DM are insulin resistance and impaired pancreatic *β* cell function [2]. With the improvement of living standards, the prevalence of T2DM has gradually increased. According to the statistics of the International Diabetes Federation (IDF), the prevalence of diabetes reached 9.3% (463 million people) in 2019. In 2021, the number of adults with diabetes in the world has reached 537 million people. The number of diabetic patients in China ranks first [3]. T2DM seriously affects the quality of life of human beings and is already a public health burden [4].

Traditional Chinese medicine has prominent advantages in the treatment of T2DM, and the curative effect is remarkable. Rhizoma Coptidis-Eupatorium fortunei medicine pair is a classic medicine pair from the Jianpixiaoke recipe founded by Professor Huang Yanqin, a famous Chinese medicine doctor [5]. Rhizoma Coptidis is bitter in taste and cold in nature; returns to the heart, spleen, stomach, liver, gallbladder, and large intestine meridians; and has the functions of clearing away heat and dampness, purging fire, and detoxifying [6]. Eupatorium fortunei is flat in nature and pungent in taste; belongs to the spleen, stomach, and lung meridians; and has the functions of aromatizing dampness, refreshing the spleen, and appetizing [7]. Rhizoma Coptidis is bitter and dry, removes dampness and hardens yin, and clears heat from turbidity, and Eupatorium fortunei is aromatic and turbid and is good at “removing stale gas” (“Su Wen: The Treatise on Strange Diseases”). The main active ingredients of Rhizoma Coptidis-Eupatorium fortunei medicine pair include luteolin, sitosterol, stigmasterol, and berberine, which has pharmacological effects such as antitumor, antihyperglycemia, antiobesity, lipid-lowering, anti-inflammatory, and improving insulin resistance [8], which is widely used in the clinic, mostly used in the treatment of diabetes, coronary atherosclerotic heart disease, etc. It has achieved a good curative effect and has great research and development potential in the future.

**Figure 1:**
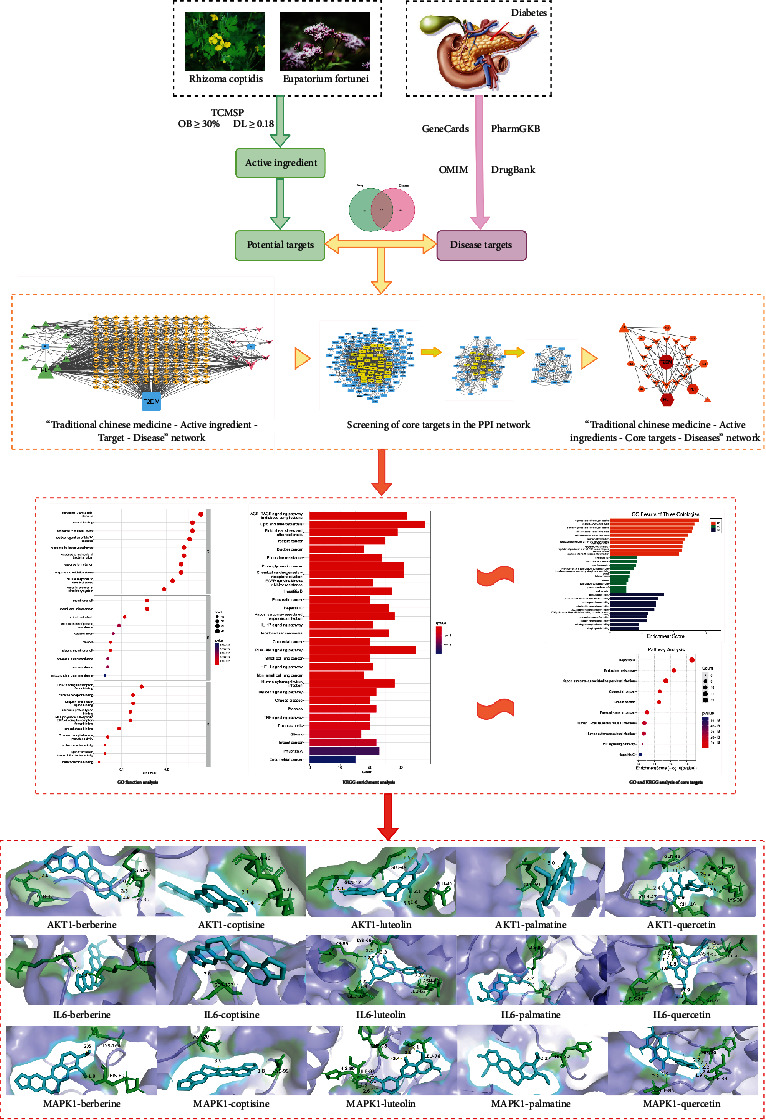
Flow chart of the mechanism of action of Rhizoma Coptidis-Eupatorium fortunei in the treatment of T2DM.

Network pharmacology is an emerging discipline integrating molecular biology, pharmacology, and bioinformatics. It analyzes the network relationship of the “drug-target-disease-pathway” to elucidate the mechanism of action of traditional Chinese medicine in treating diseases [9]. In modern times, network pharmacology has become a reasonable prediction method for the research and development of traditional Chinese medicine. Molecular docking is a technology that combines drugs with protein receptors and judges their affinity according to their binding energy [10]. The purpose of this study was to explore the components, targets, and pathways of Rhizoma Coptidis-Eupatorium fortunei on T2DM by using network pharmacology and molecular docking research methods, in order to provide a reference for the further development of the Rhizoma Coptidis-Eupatorium fortunei drug pair. The specific mechanism is shown in Figure 1.

## 2. Materials and Methods

### 2.1. Collection and Screening of Active Ingredients of R-E Drugs

Using “Huanglian” and “Peilan” as the keywords, respectively, the data of R-E drug pairs were obtained through the Traditional Chinese Medicine System Pharmacology Database and Analysis Platform (TCMSP) (https://old.tcmsp-e.com/tcmsp.php) [9]. Active chemical constituents, based on oral bioavailability (OB) and drug-like (DL), and potentially active constituents were screened out. Eligible candidate compounds were screened with OB ≥ 30% and DL ≥ 0.18.

### 2.2. Target Screening of R-E Drugs for Active Ingredients

The targets corresponding to the active ingredients in the R-E drug pair were collected in TCMSP. Since the targets provided by TCMSP may be incomplete, by using the structural information of the active components obtained from PubChem (https://pubchem.ncbi.nlm.nih.gov/), this study also developed two similarity-based target prediction networks. Targets, namely, Swiss Target Prediction (http://www.swisstargetprediction.ch/) and STITCH (http://stitch.embl.de/), were retrieved on the server to complement the targets of active ingredients in R-E drug pairs. Finally, through the UniProt (https://www.uniprot.org/) database, the research species is limited to “human,” and the screened targets are converted into corresponding standard genes.

### 2.3. Prediction and Screening of Disease Targets

Take “type 2 diabetes mellitus” as the search keyword in the GeneCards database (https://www.genecards.org/), OMIM database (https://www.omim.org/), DrugBank database (https://go.drugbank.com/), and PharmGKB database (https://www.pharmgkb.org/) to select potential targets of T2DM. The higher the score in the GeneCards database, the stronger the correlation between the target and disease. Therefore, the target with a score ≥ 30 in the GeneCards database is set as the T2DM target. After merging 4 disease database targets, the duplicate values are deleted as disease candidate targets, and the resulting targets are merged. Query the gene name of the target in the UniProt database, and standardize the candidate target.

### 2.4. Screening Drug Pair-T2DM Common Targets and Building a Network of “Traditional Chinese Medicine-Active Ingredient-Target-Disease”

The potential targets of R-E drug pairs screened in 2.2 were intersected with the disease-related targets obtained in 2.3, and a Venn diagram was drawn. The obtained R-E drug pair-T2DM intersection targets, corresponding active ingredients, traditional Chinese medicines, and disease were input into the Cytoscape 3.9.0 software to construct a “Traditional Chinese medicine-active ingredient-target-disease” network.

### 2.5. Construction of Protein-Protein Interaction (PPI) Network and Screening of Core Targets

The intersection target of R-E drug pair-T2DM obtained in 2.4 was imported into the STRING database (https://string-db.org/) to construct a common target protein-protein interaction (PPI) network. The biological species were set as “Homo sapiens,” the minimum confidence is 0.900, and the rest of the parameters are default settings to build a PPI network. And import the constructed PPI graph into Cytoscape 3.9.0 for topology analysis. The topological properties of nodes were evaluated using 6 parameters in App-Cyto NCA: Degree Centrality (DC), Betweenness Centrality (BC), Closeness Centrality (CC), Eigenvector Centrality (EC), Network Centrality (NC), and Local Average Connectivity (LAC). The higher the DC, BC, CC, EC, NC, and LAC values of each node, the more important it is in the network. Based on the topological analysis results of PPI, the target above the median was selected as the core target and screened twice, and the screened target was the core target of R-E in the treatment of T2DM.

### 2.6. GO and KEGG Enrichment Analyses

Use the R 4.1.2 software to install the relevant plug-ins from the Bioconductor website. Convert symbols of “active ingredient-disease” common targets to IDs, and perform GO enrichment analysis and KEGG pathways for common targets. Enrichment analysis was performed, the obtained results were visualized, and bar charts and bubble charts were drawn, in order to analyze the possible mechanism of R-E drugs in the treatment of T2DM from the perspective of biological functions and signaling pathways. With *p* < 0.05 as the screening condition, the most significant KEGG enrichment pathway was screened.

### 2.7. Molecular Docking

Molecular docking of important targets with key chemical components. The small molecule ligand file was downloaded from the PubChem database, imported into the Chem3D software for optimization, and then processed by the AutoDockTools software to save the small molecule in pdbqt format. The gene ID of the important target was retrieved through the UniProt database, and the three-dimensional structure of the protein receptor was obtained in the PDB database (https://www.rcsb.org/). The obtained macromolecular receptor file was imported into the AutoDockTools software for dehydration and hydrogenation and saved in pdbqt format. Finally, essential targets and active ingredients were imported into the AutoDockTools software for molecular docking, and binding energy was used as the docking evaluation index. Three representative groups of ligands and receptors were selected for data visualization processing using Pymol software.

## 3. Results

### 3.1. Screening of Chemical Components of R-E Drug Pair

The TCMSP database was searched with OB ≥ 30% and DL ≥ 0.18 as the criteria, and 25 active components were finally obtained, including 14 active components from 48 components of Rhizoma Coptidis and 11 active components from 60 components of Eupatorium fortunei. After one-to-one correspondence between chemical components and potential targets, 19 effective chemical components of Rhizoma Coptidis and Eupatorium fortunei were obtained. The UniProt database was used to convert the obtained target names into standard gene names, and 452 R-E drug pair targets were obtained after weight removal. The effective chemical components of R-E in treating T2DM are shown in Table 1.

### 3.2. Prediction Results of R-E Drug on Potential Targets

Because the targets provided by the TCMSP database are incomplete, this study also supplemented the targets corresponding to the active components of Rhizoma Coptidis and Eupatorium fortunei in the Swiss Target Prediction database and STITCH database and standardized the targets in UniProt. Finally, 12 effective chemical components and 350 targets of Rhizoma Coptidis, 7 effective chemical components and 155 targets of Eupatorium fortunei were obtained. 284 targets were obtained after the combination and de-duplication of two drug-effective ingredient targets.

### 3.3. Screening Results of Disease Targets and Drug Pair-T2DM Intersection Targets

**Figure 2:**
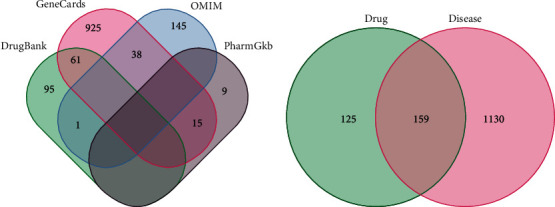
Venn diagram: (a) disease database target; (b) drug pair-T2DM intersection target.

Taking “type 2 diabetes mellitus” as the keyword, we searched the GeneCards database, OMIM database, DrugBank database, and PharmGKB database and obtained the targets of the disease: 1039 targets in the GeneCards database, 184 targets in the OMIM database, 157 targets in the DrugBank database, and 24 targets in the PharmGKB database. The obtained targets were merged, and deduplicated, and 1289 action targets related to T2DM were obtained. After Venn analysis with drug targets, 159 intersecting targets were obtained. The target information of each disease database and Venn diagram of disease and drugs are shown in Figure 2.

### 3.4. Network Construction Results of “Traditional Chinese Medicine-Active Ingredient-Target-Disease”

**Figure 3:**
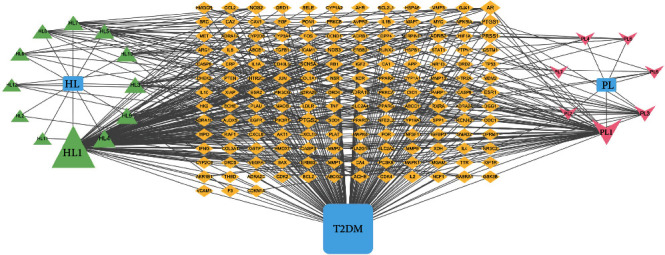
“Traditional Chinese medicine-active ingredient-target-disease” network. In this network, HL represents Rhizoma Coptidis, PL represents Eupatorium fortunei, the green triangle represents the effective active components of Rhizoma Coptidis, the pink V represents the effective active components of Eupatorium fortunei, the orange diamond represents the intersection target of R-E drug pair and T2DM, the area of the node represents its degree value, and the node area indicates the importance of the node.

The “Traditional Chinese medicine-active ingredient-target-disease” multidirectional network between R-E drug pair and T2DM was constructed by using the Cytoscape 3.9.0 software. The results show that the network consists of 181 nodes and 504 edges, including 19 R-E drug pair chemical composition nodes and 159 intersection target nodes. Taking 2 times greater than the median node degree value as the screening condition, it is concluded that quercetin, luteolin, berberine, hydrogenated berberine ((R)-canadine), palmatine, and stigmasterol are the top 6 chemical components with degree values of 126, 58, 25, 18, 17, and 17. These chemical components may play a key role in the treatment of T2DM with R-E drugs. The network of “Traditional Chinese medicine-active ingredient-target-disease” is shown in Figure 3.

### 3.5. PPI Network Construction and Core Target Screening Results

**Figure 4:**
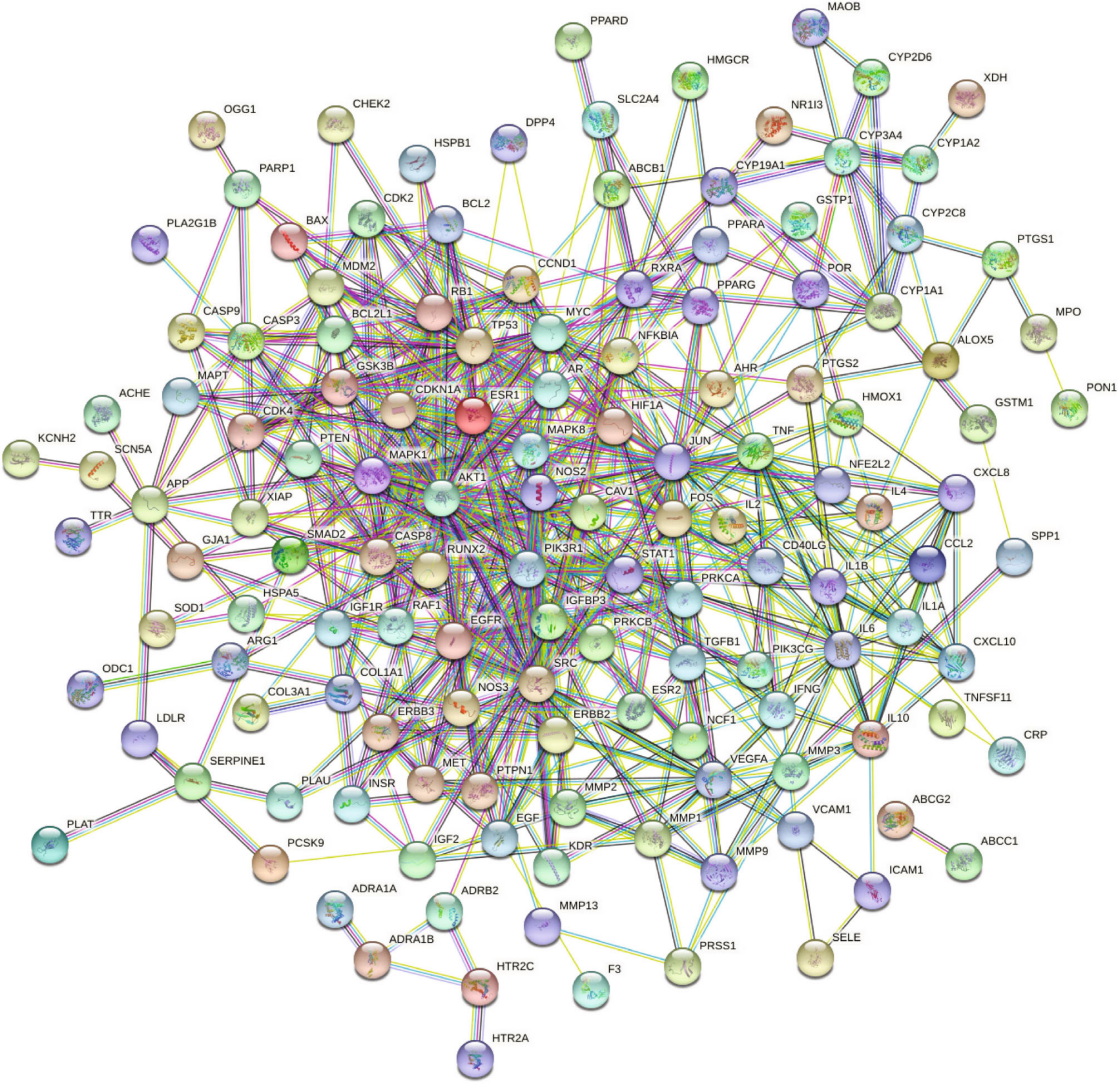
PPI network.

Import the intersection target of the R-E drug pair and T2DM into the STRING database for PPI analysis, limit the species to “Homo sapiens,” set the minimum confidence to 0.900, hide the free points, and construct the PPI network. The results show that the network contains 159 nodes and 589 edges, and the average node degree is 7.41. The PPI network is shown in Figure 4.

**Figure 5:**
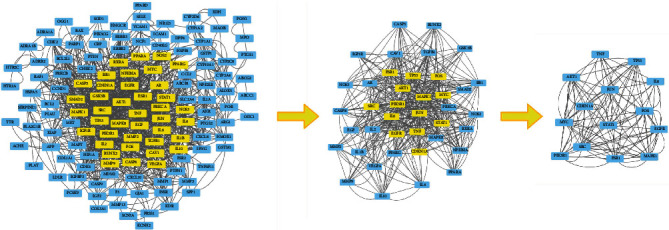
Core target screening.

Download the TSV file, and import it into the Cytoscape 3.9.0 software. Apply the cyto NCA plug-in to calculate the BC, CC, DC, EC, LAC, and NC values of each node, and conduct a topological analysis of the results. Finally, the top 14 targets with the largest correlation are obtained, which are JUN, MAPK1, SRC, AKT1, PIK3R1, TP53, TNF, FOS, STAT1, ESR1, IL6, CDKN1A, EGFR, and MYC. These targets may be the core targets of the R-E drug pair for the treatment of T2DM. The core network screening process is shown in Figure 5.

**Figure 6:**
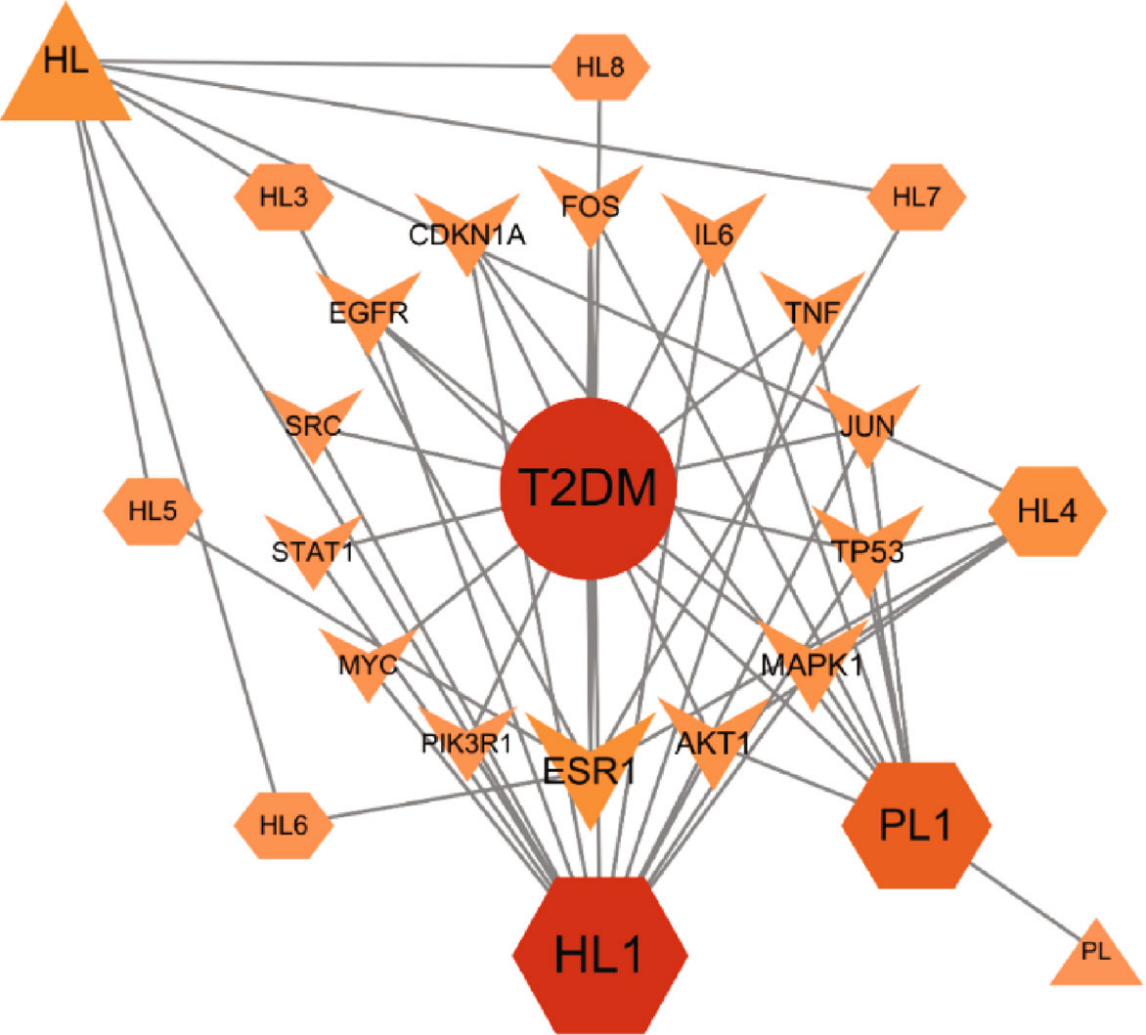
“Traditional Chinese medicine-active ingredient-core target-disease” network.

The screened core targets, important chemical components, Chinese herbal medicine, and disease are introduced into Cytoscape 3.9.0 to obtain the “Traditional Chinese medicine-active component-core target-disease” network, so as to better screen the core targets and important chemical components of R-E drugs for the treatment of T2DM. The network consists of 25 nodes and 53 sides. The results show that the core targets are ESR1, MAPK1, AKT1, TP53, IL6, and JUN in turn. The important chemical components are HL1 (quercetin), PL1 (luteolin), HL4 (berberine), HL3 (palmatine), and HL5 (coptisine). The network of “Traditional Chinese medicine-active ingredient-core target-disease” is shown in Figure 6.

### 3.6. Enrichment Analysis Results

**Figure 7:**
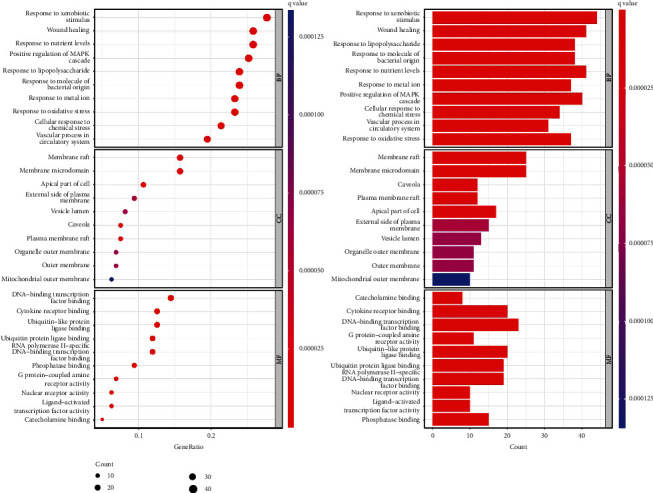
Biological function enrichment map of the top 30 GO.

In GO enrichment analysis, the results obtained 2960 GO entries, including 2660 biological process (BP) entries, 95 cell composition (CC) entries, and 205 molecular function (MF) entries (the selection condition is*p* < 0.05). Since the *p* value represents the significance of enrichment, the first 10 items with the lowest *p* value among BP, CC, and MF were selected, and the bar graph and bubble graph of GO biological function enrichment were made by using the R 4.1.2 software. The GO analysis results are shown in Figure 7.

The results showed that the BP of the intersection target of the R-E drug pair and T2DM was mainly concentrated in response to xenobiotic stimulus, wound healing, response to nutrient levels, positive regulation of MAPK cascade, and response to lipopolysaccharide; CC is mainly concentrated in membrane raft, membrane microdomain, apical part of cell, external side of plasma membrane, vesicle lumen, etc. MF mainly focuses on DNA-binding transcription factor binding, cytokine receptor binding, ubiquitin-like protein ligase binding, ubiquitin protein ligase binding, RNA polymerase II-specific DNA-binding transcription factor binding, etc.

**Figure 8:**
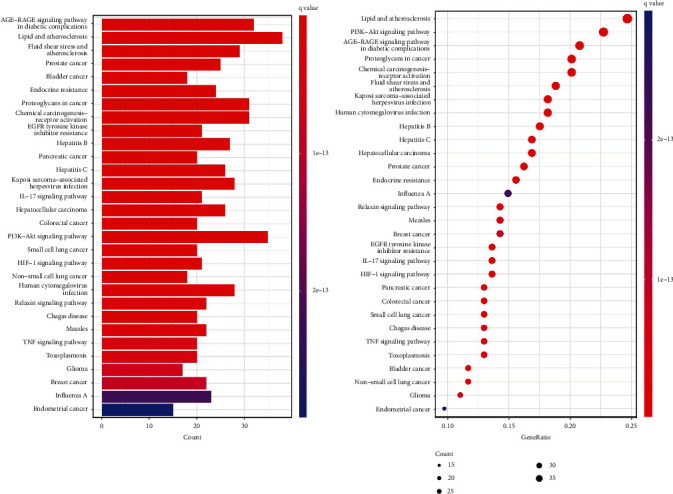
Enrichment analysis of the first 30 KEGG pathways.

Using KEGG pathway enrichment analysis, 182 KEGG signal pathways were obtained (*p* < 0.05). Due to the enrichment significance of the *p* value table, the first 30 signal channels with the lowest *p* value are selected, and the KEGG signal channel enrichment bar graph and bubble graph are made by using the R 4.1.2 software. KEGG analysis results are shown in Figure 8. The results showed that the signaling pathways of R-E drugs on the intersection targets with T2DM mainly involved lipid and atherosclerosis, PI3K-Akt signaling pathway, AGE-RAGE signaling pathway in diabetic complications, proteoglycans in cancer, chemical carcinogenesis-receptor activation, fluid shear stress and atherosclerosis, Kaposi's sarcoma-associated herpesvirus infection, human cytomegalovirus infection, hepatitis B, hepatitis C, hepatocellular carcinoma, prostate cancer, endocrine resistance, influenza A, relaxin signaling pathway, measles, breast cancer, EGFR tyrosine kinase inhibitor resistance, IL-17 signaling pathway, HIF-1 signaling pathway, pancreatic cancer, colorectal cancer, etc.

**Figure 9:**
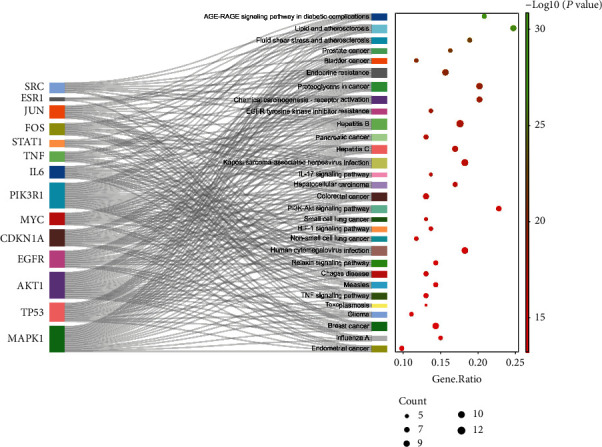
Enrichment information of core targets in important pathways.

This study screened the number of core targets enriched in the key KEGG pathway and further analyzed the enriched pathways. It was found that hepatitis B, Kaposi's sarcoma-associated herpesvirus infection, endocrine resistance, proteoglycans in cancer, human cytomegalovirus infection, and breast cancer pathway contained more than 10 core targets, which showed that the relationship between cancer, inflammation, and T2DM was inseparable, Whether there is a close relationship within its mechanism needs further study. The specific enrichment pathways of core targets are shown in Figure 9.

**Figure 10:**
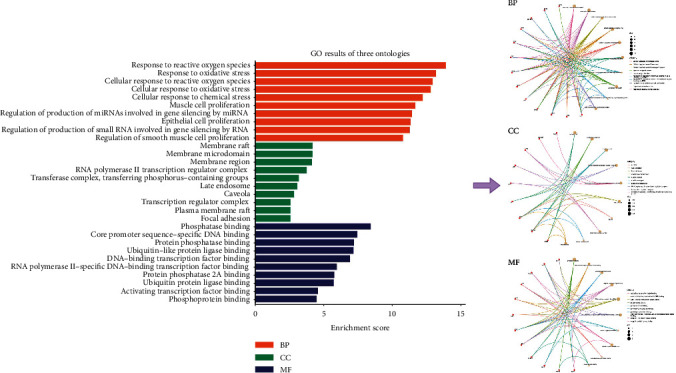
Biological function enrichment map of the top 30 GO.

**Figure 11:**
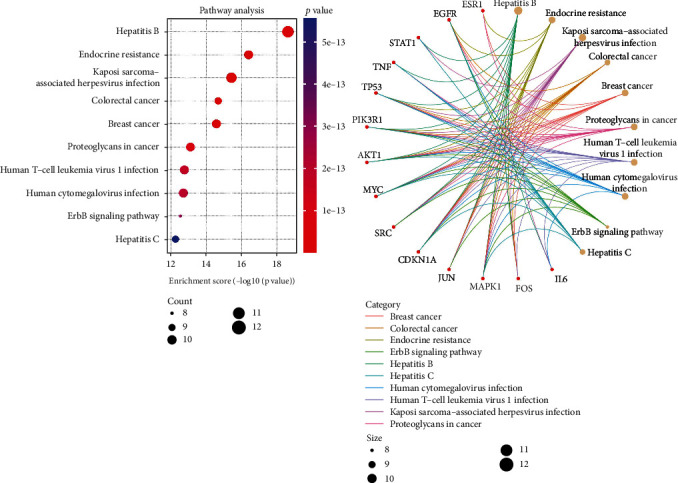
Enrichment analysis diagram of the top 10 KEGG pathways.

This study also conducted GO and KEGG analyses on the core targets of R-E drugs for the treatment of T2DM. A total of 1670 entries were obtained from the enrichment results of GO, including 1531 entries for BP, 41 entries for CC, and 98 entries for MF. GO analysis of core target is shown in Figure 10. A total of 148 pathways were enriched by KEGG pathway enrichment analysis. KEGG analysis of core target is shown in Figure 11. The analysis results show that the core targets are mainly concentrated in hepatitis B (hsa05161), endocrine resistance (hsa01522), Kaposi's sarcoma-associated herpesvirus infection (hsa05167), colorectal cancer (hsa05210), breast cancer (hsa05224), proteoglycans in cancer (hsa05205), and other pathways, which are roughly the same as the enrichment of drug pair-T2DM intersection targets. It can be seen that the core targets selected in this study are representative.

### 3.7. Molecular Docking Results

**Figure 12:**
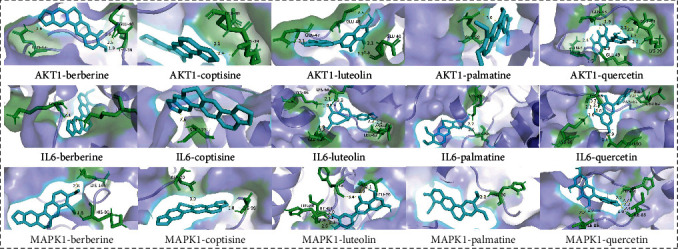
Molecular docking results.

The first 6 core targets and 5 important chemical components selected were used as receptors and ligands for molecular docking. The higher the absolute value of binding energy, the more stable the binding between receptor and ligand. The binding energy of -5.0 kcal/mol is set as the threshold for whether the receptor and ligand bind well. Berberine, palmatine, and coptisine have high binding to core targets. MAPK1, AKT1, and IL6 interact well with these key components. This shows that some effective components in R-E drug pairs have good binding properties with protein targets. After comprehensive consideration, the representative molecular docking results are visualized, see Figure 12 for details. All molecular docking results are shown in Table 2.

## 4. Discussion

The previous research of our research group found that Jianpixiaoke recipe can significantly improve the number of rat islets *β* cells, and their distribution in islets, mediate endoplasmic reticulum stress, promote autophagy, inhibit apoptosis, improve basal and glucose stimulated insulin secretion, and protect islets *β* Cell number and function [11]. Rhizoma Coptidis-Eupatorium fortunei is a classic medicine pair in the Jianpixiaoke recipe, which has the effect of invigorating the spleen and clearing away heat.

The pathogenesis of T2DM is complex, and there are many important compound components, targets, and pathways, but the mechanism of Rhizoma Coptidis and Eupatorium fortunei in the treatment of T2DM has not been fully clarified. In order to further explore the material basis and possible mechanism of its improvement in patients with T2DM, this study systematically predicted the potential chemical components, key targets, molecular mechanisms, and related pathways of Rhizoma Coptidis and Eupatorium fortunei in the treatment of T2DM through the research methods of network pharmacology and molecular docking. After searching multiple databases, a total of 19 chemical components and 284 targets of R-E drug pair, 1289 T2DM-related targets, 159 common targets of chemical components of drug pair and disease, and 182 potential signal pathways were screened. These results reflect the potential anti-inflammatory mechanism of Rhizoma Coptidis and Eupatorium fortunei in the treatment of T2DM.

Quercetin reduces the effect on pancreatic islets through anti-inflammatory and antioxidant effect *β* cell damage, promotes insulin secretion, and regulates blood glucose balance [12]. Quercetin downregulates the expression of Wnt3a and p-GSK-3*β*, and *β*-catenin protein can reduce the level of oxidative stress in renal tissue and protect the renal function of diabetic rats [13]. Luteolin has an obvious hypoglycemic effect, improving insulin resistance in DM rats by downing the expression of TLR4, JUK mRNA, and protein levels [14]. Other studies have found that luteolin is inhibited by a reversible process in a noncompetitive manner the catalytic activity of *α*-glucosidase [15], significantly reduces blood glucose in T2DM rats, improves the level of glycosylated hemoglobin in the body, and reduces insulin resistance [16]. Berberine can participate in glucose and lipid metabolism by upregulating the levels of glucagon-like peptide-1 (GLP-1) and ghrelin in the intestine, so as to achieve the purpose of regulating blood glucose [17, 18]. Palmatine has anti-inflammatory, antibacterial, antihypertensive, anticancer, and other pharmacological effects [19]. Li et al. [20] found that palmatine can effectively improve the fasting blood glucose and blood lipid levels of db/db diabetes mice, inhibit oxidative stress, and reduce the impact on islet *β* cellular damage. Coptisine can reduce blood glucose by promoting the transport and absorption of glucose [21].

This study also revealed that ESR1, MAPK1, AKT1, TP53, IL6, and JUN may be the potential key targets of R-E in the treatment of T2DM. ESR1 and islet *β* apoptosis are closely related, and it is also one of the biological indicators for clinical diagnosis of gestational diabetes in early pregnancy [22]. MAPK1 can reduce blood glucose levels in patients with DM, improve oxidative stress, and protect pancreatic *β* cells by regulating MAPK/ERK cascade reaction [23]. AKT1 is an important target downstream of insulin signaling pathway, which can regulate glucose transport and blood glucose level by blocking insulin receptor dephosphorylation [24]. As a tumor suppressor gene, TP53 is closely related to metabolism and can affect insulin resistance in the body [25]. IL6 is an inflammatory factor, which can increase insulin resistance by upregulating the expression of cytokine signal pathway inhibitor 3 [26], while blocking IL6 signal transduction can effectively improve glucose metabolism and insulin sensitivity. Luteolin in Eupatorium fortunei can play an anti-inflammatory and antioxidant role, reduce the secretion of IL6, and reduce the expression of inflammatory factors [27]. JUK is activated, which can downregulate insulin signal transduction, reduce the sensitivity of surrounding tissue cells to insulin, and lead to glucose and lipid metabolism disorders [28].

The results of GO functional enrichment analysis suggest that R-E can improve T2DM by regulating a variety of BP and MF, which may interfere with T2DM through biological processes such as glucose homeostasis and lipopolysaccharide reaction. These targets are mainly located in the plasma membrane and its components. In the KEGG enrichment analysis results of this study, the key targets are mainly enriched in AGE-RAGE, endocrine resistance, PI3K-Akt, IL-17, HIF-1, TNF, and other signal pathways. Among them, the pathways involved in glucose and lipid metabolism and insulin resistance include AGE-RAGE, PI3K-Akt, and endocrine resistance. As an important link in the generation and development of microvascular complications of diabetes, the AGE-RAGE signaling pathway regulates the process of impaired islet cell function [29]. Berberine can significantly reduce blood glucose in rats with diabetic nephropathy, and its mechanism of protecting renal function may be related to downregulation of AGEs-RAGE signal pathway [30]. Insulin resistance can downregulate PI3K-Akt pathway, which plays an important role in insulin regulation by regulating growth factors, reducing insulin resistance, enhancing glucose transport function, and regulating blood glucose and lipid metabolism [31]. Berberine can improve renal function by downregulating the expression of PI3K and AKT [32]. IL-17 signaling pathway is closely related to T2DM and can increase local cytokines, aggravate the inflammatory response of islets, and induce islet *β* apoptosis [33]. Studies show that IL-17 can activate NF-*κ*B pathway, upregulate TNF-*α* expression, and induce insulin resistance [34]. HIF-1 signaling pathway plays an important role in many pathophysiological processes, such as angiogenesis, cell proliferation, migration, apoptosis, and energy metabolism. Some studies have shown that the local hypoxic microenvironment after trauma can activate nuclear factors through PI3K/AKT signaling channel *Κ*B expression, leading to HIF-1*α* increased expression [35]. HIF-1 signaling pathway can upregulate the expression of VEGF, downregulate the expression of interleukin-10, and increase inflammation [36]. Quercetin can downregulate HIF-1*α*. It can increase the expression level of p53, improve the hypoxia state of islet cells, and increase the sensitivity of surrounding cells to insulin. As a classic pathway of inflammatory response, TNF signaling pathway is closely related to the occurrence of DM [37]. TNF-*α* is an important inflammatory factor, and its elevated level can interfere with insulin signal transduction. At the same time, TNF-*α* binding with cell surface receptors can affect the phosphorylation of insulin receptors and disorder blood glucose metabolism [38].

In this study, through GO and KEGG analyses of the target and core target of R-E in the treatment of T2DM, it is found that most of the enriched pathways are related to cancer and inflammation. Now, epidemiological studies show that diabetes can induce the incidence and mortality of pancreatic cancer, colorectal cancer, and other tumors [39]. Although the mechanism of the two is not completely clear, the relationship between the two is indeed closely related. T2DM often leads to a variety of metabolic disorders, such as hyperinsulinemia, hyperlipidemia, hyperglycemia, and inflammation, which may induce the risk of cancer [40, 41]. Hepatitis B infection can induce T2DM or increase the risk of T2DM. Ji et al. reported that HBV preS2 protein can reduce the expression of INSR by reducing the activity of insulin receptor gene promoter, resulting in the decrease of insulin sensitivity [42]. These studies suggest the feasibility of the target and signal pathway predicted in this study. It has been proved that luteolin can downregulate TNF-*α* and IL6, thereby increasing insulin [43]. MAPK signaling pathway can downregulate the expression of glucose transporter 4 and increase blood glucose concentration by reducing glucose transport. Studies have shown that Rhizoma Coptidis can downregulate MAPK signal pathway, upregulate the expression of glucose transporter 4, and effectively reduce blood glucose [44].

Molecular docking results showed that berberine, palmatine, and coptisine had high binding activity to the core target. Among them, MAPK1, AKT1, and IL6 have better docking results with these effective active ingredients. This suggests that berberine, palmatine, and coptisine may be the main active components of Rhizoma Coptidis-Eupatorium fortunei against T2DM.

## 5. Conclusion

Through the network pharmacology and molecular docking technology, this study confirmed that Rhizoma Coptidis-Eupatorium fortunei may treat T2DM through anti-inflammatory and regulating glucose and lipid metabolism. Network pharmacology and molecular docking have revealed the mechanism of traditional Chinese medicine in the treatment of T2DM at the molecular level, which provides an effective basis for Rhizoma Coptidis-Eupatorium fortunei in the clinical treatment of T2DM. However, at present, network pharmacology is still in the development stage, the computing software and related database layers are different, each has its own characteristics, and the data about various drugs, targets, and diseases are not comprehensive. Therefore, further experimental verification and more rigorous research strategies should be carried out in the future. The research team will continue to carry out further experimental validation and clinical application of Rhizoma Coptidis-Eupatorium fortunei and continue to explore the potential of traditional Chinese medicine in the treatment of T2DM.

## Figures and Tables

**Table 1:** Effective chemical components of R-E in the treatment of T2DM.

Effective chemistry component code	Molecular name	OB (%)	DL	Drug source
MOL001454	Berberine	36.86	0.78	Rhizoma Coptidis
MOL013352	Obacunone	43.29	0.77	Rhizoma Coptidis
MOL002894	Berberrubine	35.74	0.73	Rhizoma Coptidis
MOL002897	Epiberberine	43.09	0.78	Rhizoma Coptidis
MOL002903	(R)-Canadine	55.37	0.77	Rhizoma Coptidis
MOL002904	Berlambine	36.68	0.82	Rhizoma Coptidis
MOL002907	Corchoroside A_qt	104.95	0.78	Rhizoma Coptidis
MOL000622	Magnograndiolide	63.71	0.19	Rhizoma Coptidis
MOL000762	Palmidin A	35.36	0.65	Rhizoma Coptidis
MOL000785	Palmatine	64.60	0.65	Rhizoma Coptidis
MOL000098	Quercetin	46.43	0.28	Rhizoma Coptidis
MOL001458	Coptisine	30.67	0.86	Rhizoma Coptidis
MOL002668	Worenine	45.83	0.87	Rhizoma Coptidis
MOL008647	Moupinamide	86.71	0.26	Rhizoma Coptidis
MOL000006	Luteolin	36.16	0.25	Eupatorium fortunei
MOL000359	Sitosterol	36.91	0.75	Eupatorium fortunei
MOL000363	Amyrin palmitate	32.68	0.30	Eupatorium fortunei
MOL000449	Stigmasterol	43.83	0.76	Eupatorium fortunei
MOL000584	7-Acetoxy-8-hydroxy-9-isobutyryloxythymol	33.39	0.18	Eupatorium fortunei
MOL000588	9-Acetoxy-8,10-epoxy-6-hydroxythymol 3-O-angelate	61.44	0.21	Eupatorium fortunei
MOL000592	Dammaradienyl acetate	46.52	0.82	Eupatorium fortunei
MOL000595	Eupatoriopicrin	76.78	0.36	Eupatorium fortunei
MOL000596	[(3S,4aR,6aR,6aR,6bR,8aR,12S,12aR,14aR,14bR)-4,4,6a,6b,8a,12,14b-Heptamethyl-11-methylene-1,2,3,4a,5,6,6a,7,8,9,10,12,12a,13,14,14a-hexadecahydropicen-3-yl] acetate	43.08	0.74	Eupatorium fortunei
MOL000604	Eupaformosanin	50.20	0.52	Eupatorium fortunei
MOL000605	Taraxasterol palmitate	33.84	0.31	Eupatorium fortunei

**Table 2:** Molecular docking results of core targets and key active ingredients.

Hub targets	Quercetin (kcal/mol)	Luteolin (kcal/mol)	Berberine (kcal/mol)	Palmatine (kcal/mol)	Coptisine (kcal/mol)
ESR1	-4.99	-4.82	-7.72	-8.05	-8.33
MAPK1	-5.35	-5.20	-8.14	-8.04	-8.25
AKT1	-6.37	-6.54	-8.72	-8.28	-8.95
TP53	-4.04	-4.52	-6.35	-6.26	-6.59
IL6	-6.48	-6.37	-8.39	-8.07	-8.53
JUN	-4.45	-4.90	-9.22	-8.78	-9.58

## Data Availability

The data used to support the findings of this study are available from the corresponding author upon request.
